# Sodium Silicate/Urea/Melamine Ternary Synergistic Waterborne Acrylic Acid Flame-Retardant Coating and Its Flame-Retardant Mechanism

**DOI:** 10.3390/molecules29071472

**Published:** 2024-03-26

**Authors:** Yuran Shao, Yuting Wang, Fei Yang, Chungui Du, Jiawei Zhu, Ying Ran, Qichao Bao, Yingying Shan, Weigang Zhang

**Affiliations:** 1 Bamboo Industry Institute, Zhejiang A & F University, Hangzhou 311300, China; shao18309819091@163.com (Y.S.); wang270229@163.com (Y.W.); yangfeier0826@163.com (F.Y.); ry18119295807@163.com (Y.R.); bqc1125573308@gmail.com (Q.B.); 20091018@zafu.edu.cn (W.Z.); 2 College of Chemistry and Materials Engineering, Zhejiang A & F University, Hangzhou 311300, China; syy15968566686@163.com

**Keywords:** sodium silicate, ternary synergistic flame retardant, flame-retardant coating, flame-retardant mechanism, poplar veneer

## Abstract

Waterborne acrylic coatings, the largest market share of predominant environmentally friendly coatings, face limitations in their extensive application due to their flammability. The flame-retardant properties of the coatings could be significantly enhanced by incorporate inorganic flame retardants. However, inorganic flame retardants tend to aggregate and unevenly disperse in waterborne acrylic coatings, causing a substantial decrease in flame retardancy. In this work, sodium silicate was utilized as a flame retardant, with urea and melamine serving as modifiers and synergistic agents. This combination resulted in the preparation of a sodium silicate/urea/melamine ternary synergistic waterborne acrylic flame-retardant coating. This coating was applied to the surface of poplar veneer to create flame-retardant poplar veneer. Subsequently, various instruments, including a scanning electron microscope (SEM), a limiting oxygen index meter (LOI), a thermogravimetric analyzer (TG), and a cone calorimeter (CONE), were employed to investigate the relevant properties and mechanisms of both the flame-retardant coating and poplar veneer. The results demonstrated that the sodium silicate/urea/melamine ternary synergistic flame retardant did not exhibit aggregation and could be uniformly dispersed in waterborne acrylic coatings. The physical and mechanical properties of the ternary synergistic flame-retardant poplar veneer coating were satisfactory. Melamine and urea, acting as modifiers, not only greatly enhanced the dispersibility of sodium silicate in waterborne acrylic coatings, but also assisted in the formation of a silicon-containing char layer through the generation of nitrogen, achieving ternary synergistic flame retardancy. In conclusion, this work explores a novel method to efficiently and uniformly disperse inorganic flame retardants in organic coatings. It significantly improves the dispersibility and uniformity of inorganic flame retardants in organic polymers, thereby substantially enhancing the flame-retardant performance of coatings. This work provides a theoretical basis for the research and application of new flame-retardant coatings in the field of chemistry and materials.

## 1. Introduction

Waterborne coatings, also known as water-based paint or waterborne paint, represent a category of coatings utilizing water as the primary solvent. They boast attributes such as non-toxicity, environmental friendliness, absence of odor, and minimal volatile organic compounds [1,2]. Presently, waterborne coatings command a 57% share of the global coating market [3], establishing themselves as a pivotal direction in the world’s coating development. Within the realm of waterborne coatings, waterborne acrylic coatings claim the highest market share and have found extensive applications across industries such as automotive, furniture, and construction [4,5,6]. However, the intrinsic flammability of waterborne acrylic coatings [7] poses challenges, as they are susceptible to ignition and combustion, resulting in building fires and significant safety incidents. This constraint hampers the widespread adoption of waterborne acrylic coatings [8]. Consequently, investigating flame-retardant waterborne acrylic coatings assumes paramount significance. Despite this, research reports on flame-retardant waterborne acrylic coatings remain relatively scarce, necessitating further systematic exploration.

The predominant approach to fabricating flame-retardant waterborne acrylic coatings involves incorporating flame retardants into the coatings. Flame retardants are categorized into inorganic and organic types based on their chemical composition [9], with inorganic flame retardants being more extensively researched and applied. Among inorganic flame retardants, silicon-based flame retardants, with their commendable flame-retardant performance, straightforward processing, and cost-effectiveness, exhibit vast application prospects. Research reports on the preparation of flame-retardant coatings using silicon-based flame retardants such as sodium silicate, nano-SiO_2_, and layered silicate have been published [10,11,12,13]. However, the formation pathways of inorganic-phase silicon-based flame-retardants and organic component coatings markedly differ. Silicon-based flame retardants tend to dry, crystallize, and unevenly aggregate within the coating, hindering the formation of a uniform thermal insulation coating on the material’s surface. This, in turn, leads to a substantial decrease in the flame-retardant performance of the coating [14,15,16].

Wood veneer is a type of wood processing material widely used in the fields of construction, furniture, musical instruments, and more. Its excellent processing performance and aesthetic appeal allow it to be crafted into various shapes and sizes of products, such as veneer materials for flooring, wall panels, doors, tables, etc. [17,18]. Despite the aesthetic appeal, durability, and stability of wood veneer, its flammability poses fire risks in construction and furniture manufacturing, limiting its development in everyday life. Currently, there are numerous studies on improving the physical and flame-retardant properties of wood veneer using silicon-based materials [19,20,21,22]. For instance, to enhance the dimensional stability of wood, Unger et al. [23] customized sol–gel synthesis to produce precursor species with nano-silica, which can penetrate into the cell walls of wood. In order to overcome the common issue of aggregation in silicon-based flame retardants and flame-retardant coatings, R. Werner et al. [24] found that the relatively high hydrophilicity of sodium silicate–aluminum and the low chemical affinity between silicates and the functional organic groups present in multi-component coating components are the main reasons for the difficulty of dispersing silicate aggregation. Researchers have reduced or eliminated the hydrophilicity of silicates by introducing organic functional groups, enhancing their hydrophobicity and improving the performance of sodium silicate–aluminum coatings. Inorganic silicon-based flame-retardant coatings also include nano-SiO_2_, layered silicates, and cage-like oligomeric silsesquioxane (POSS) [10,11,12,13]. Among them, coatings made of nano-SiO_2_ are prone to agglomeration or secondary aggregation due to the large number of active hydroxyl groups on their surface, resulting in poor dispersion in materials and thus affecting their structure and properties [25]. Therefore, surface modification of nano silica is required in order to ensure its stable storage and improve its dispersion [26,27]. However, uniformly incorporating silicon-based flame retardants into coatings, especially for flammable materials such as wood veneer, remains a significant challenge which requires further research on the modification of silicon-based flame retardants to overcome the difficulty of uneven dispersion and easy aggregation.

In response, this work initially modifies the flame-retardant sodium silicate with melamine and urea as modifiers, disrupting and inhibiting the recrystallization process of sodium silicate. This modification significantly enhances the uniform dispersion of sodium silicate in waterborne acrylic coatings. Subsequently, a flame-retardant waterborne acrylic coating containing sodium silicate/urea/melamine ternary synergies is prepared. Finally, the coating is applied to poplar veneer to fabricate flame-retardant poplar veneer. The flame-retardant performance and mechanism are studied. This research offers a novel perspective for improving the uniform dispersion of inorganic flame retardants in organic coatings, bearing significant implications for the development of high-performance flame-retardant waterborne acrylic coatings and their widespread adoption.

## 2. Results and Discussion

### 2.1. Appearance and Viscosity of Coatings

**Figure 1:**
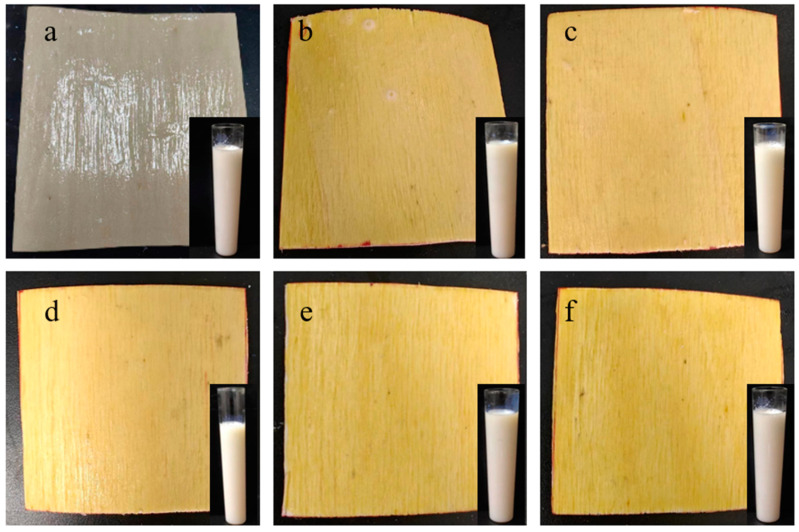
Appearance morphology of non-flame-retardant and flame-retardant coatings, and non-flame-retardant and flame-retardant poplar veneer. (**a**) A and A@V; (**b**) AS and AS@V; (**c**) ASM_7_U_3.5_ and ASM_7_U_3.5_@V; (**d**) ASM_7_U_7_ and ASM_7_U_7_@V; (**e**) ASM_7_U_10.5_ and ASM_7_U_10.5_@V; (**f**) ASM_7_U_14_ and ASM_7_U_14_@V.

By documenting the entire process of bubbles ascending in the formatted tube, the time difference from the initiation of bubble ascent to its conclusion was calculated. Subsequently, utilizing the time–viscosity correlation table, viscosity was converted to acquire viscosity values and the appearance morphology of various ratios of coatings, A, AS, and ternary synergistic flame-retardant coatings (ASM_7_U_3.5_, ASM_7_U_7_, ASM_7_U_10.5_, ASM_7_U_14_). This encompasses their appearance on poplar veneers, specifically A@V, AS@V, and ternary synergistic flame-retardant poplar veneers (ASM_7_U_3.5_@V, ASM_7_U_7_@V, ASM_7_U_10.5_@V, ASM_7_U_14_@V), as illustrated in Figure 1.

**Figure 2:**
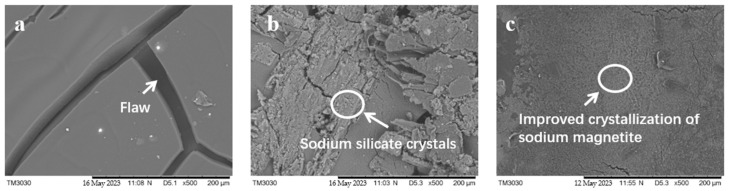
SEM images of non-flame-retardant and flame-retardant coated poplar veneers (**a**) A@V; (**b**) AS@V; (**c**) ASM_7_U_7_@V.

From Figure 1a–f, it is evident that the color of ternary synergistic flame-retardant coatings remains milky white. Therefore, the incorporation of flame retardants and modifying agents does not alter the original color of waterborne acrylic acid coatings. However, when poplar veneers are coated with sodium silicate flame-retardant coatings and ternary synergistic flame-retardant coatings, the color changes from light yellow to dark yellow. As observed in Figure 2b, there are small white crystalline particles on the surfaces of flame-retardant coatings, and after drying on poplar veneers, there is agglomeration, with some areas not evenly distributed. In Figure 1c–f, ternary synergistic flame-retardant coatings show no crystallization or agglomeration on the surface, and after drying on poplar veneers, they are evenly distributed. This suggests that the addition of melamine and urea-modifying agents in sodium silicate enhances the modification of the flame-retardant sodium silicate, improving its uniform dispersion performance and addressing the issue of crystallization and agglomeration commonly associated with silicon-based flame retardants in waterborne acrylic acid.

The dynamic viscosities of A, AS, ASM_7_U_3.5_, ASM_7_U_7_, ASM_7_U_10.5_, and ASM_7_U_14_ are 0.250 Pa·s, 0.085 Pa·s, 0.125·Pa·s, 0.125·Pa·s, 0.140·Pa·s, and 0.140 Pa·s, respectively. It can be observed that the addition of only sodium silicate to waterborne acrylic acid paint for flame-retardant coatings leads to a significant decrease in the viscosity of the coating. This phenomenon may be attributed to the high moisture content present in nonahydrate sodium silicate. Furthermore, the introduction of the modifying agents melamine and urea into waterborne acrylic flame-retardant coatings results in an increase in viscosity. Within the flame-retardant system, when the content of melamine (mass percentage) is 7%, the viscosity of sodium silicate/urea/melamine ternary synergistic flame-retardant coatings gradually increases with the rise in urea content, stabilizing at a urea content (mass percentage) of 10.5%. In comparison to non-flame-retardant waterborne acrylic coatings, ternary synergistic flame-retardant coatings exhibit lower viscosity, enhancing their suitability for coating.

### 2.2. Paint Film Hardness and Adhesion of Coatings

The paint film hardness and adhesion values of A@V, AS@V, and ternary synergistic flame-retardant poplar veneers (ASM_7_U_3.5_@V, ASM_7_U_7_@V, ASM_7_U_10.5_@V, ASM_7_U_14_@V) are presented in Table 1.

Paint film hardness, a crucial measure of coatings’ mechanical strength [28,29], indicates improved performance in wear resistance, compressive strength, and other aspects with higher values [30,31]. As depicted in Table 1, both non-flame-retardant and sodium silicate flame-retardant poplar veneers exhibited paint film hardness of HB, suggesting that the addition of sodium silicate flame-retardant in waterborne acrylic acid coatings had no impact on paint film hardness. Furthermore, the paint film hardnesses of ternary synergistic flame-retardant poplar veneers with varying ratios (ASM_7_U_3.5_@V, ASM_7_U_7_@V, ASM_7_U_10.5_@V, ASM_7_U_14_@V) were B, HB, HB, and HB, respectively. The paint film hardness remained steady at HB within the urea content range of 7% to 14%, consistent with the paint film hardness of non-flame-retardant and sodium silicate flame-retardant poplar veneers. This implies that the quantity of melamine and urea-modifying agents did not alter the paint film hardness. Given that HB falls within the moderately hard category, it signifies relatively strong strength properties in ternary synergistic flame-retardant coatings.

Additionally, Table 1 reveals that the paint film adhesion of non-flame-retardant poplar veneer, sodium silicate flame-retardant poplar veneer, and ternary synergistic flame-retardant poplar veneers with different ratios all achieved 4B ratings, meeting the requirements of ISO 1 and North American technical standard ASTM 4B. Consequently, ternary synergistic flame-retardant coatings exhibited exceptional paint film adhesion.

### 2.3. Microscopic Morphology of Coatings

Scanning electron microscope (SEM) images of A@V, AS@V, and ASM_7_U_7_@V are presented in Figure 2. The SEM analysis revealed distinct characteristics of the coatings.

From Figure 2, it is evident that the non-flame-retardant waterborne acrylic acid coating applied to the surface of poplar veneer (A@V) resulted in a uniform and smooth coating with minimal agglomeration. However, there were a few noticeable cracks present. In contrast, the sodium silicate flame-retardant coating applied to the surface of poplar veneer (AS@V) displayed a surface with a significant amount of crystalline formations and agglomerations. On the other hand, the ternary synergistic flame-retardant coating applied to the surface of the poplar veneer (ASM_7_U_7_@V) exhibited a coating surface without crystalline formations or agglomerations.

This observation indicates that the addition of melamine and urea-modifying agents had a notable positive effect on mitigating the crystalline and agglomeration issues associated with sodium silicate. Consequently, the dispersion of sodium silicate in the coating became more uniform, resulting in a more even coating surface.

### 2.4. Energy-Dispersive X-Ray Spectroscopy (EDX) Analysis

**Figure 3:**
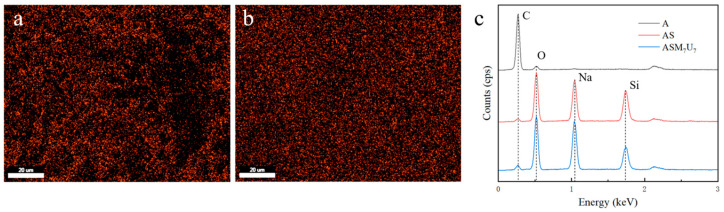
EDX spectra of coatings before and after flame retardancy. (**a**) Si element distribution in AS; (**b**) Si element distribution in ASM_7_U_7_; (**c**) EDX spectra of flame-retardant coatings.

The energy-dispersive X-ray spectroscopy (EDX) results for A, AS, and ASM_7_U_7_ are presented in Figure 3. The EDX spectra offer insights into the elemental composition before and after the flame retardancy treatment.

From Figure 3a, it is evident that the distribution of Si elements in the sodium silicate flame-retardant coating was uneven, displaying numerous bright spots with significant aggregation. Conversely, Figure 3b illustrates that the Si element distribution in the ternary synergistic flame-retardant coating was uniform, with minimal aggregation. This observation highlights the effective role of melamine and urea in mitigating the aggregation phenomenon associated with sodium silicate in the coating.

Furthermore, Figure 3c provides EDX spectra of the flame-retardant coatings, indicating that both the sodium silicate flame-retardant coating and the ternary synergistic flame-retardant coating contained the main elements C and O, characteristic of non-flame-retardant waterborne acrylic acid coating. Additionally, they both exhibited the main elements Na and Si, along with their characteristic peaks representing the composition of the sodium silicate flame retardant. However, the Si element content and characteristic peaks in the ternary synergistic flame-retardant coating were lower than those in the sodium silicate flame-retardant coating. This suggests that the addition of melamine and urea reduced the aggregation of Si elements, resulting in a more even distribution in the coating.

### 2.5. X-ray Diffraction (XRD) Analysis

**Figure 4:**
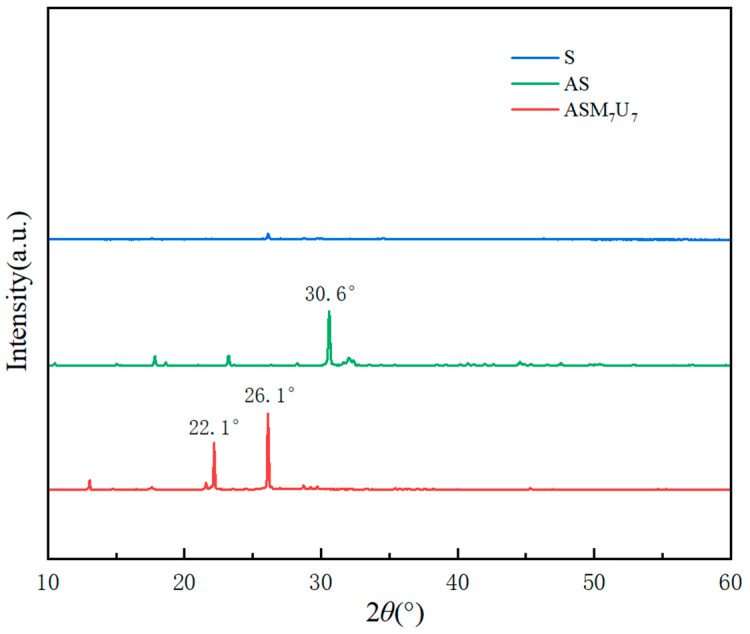
XRD patterns of non-flame-retardant and flame-retardant coatings.

In Figure 4, the X-ray diffraction (XRD) patterns unveil significant insights into the structural characteristics of S, AS, and ASM_7_U_7_. The XRD pattern of sodium silicate solution (S) reveals relatively smooth and indistinct characteristic peaks, attributed to the strong hydrophilicity of the hydroxyl groups in sodium silicate. These hydroxyl groups contribute to its excellent water solubility. On the other hand, AS exhibits a pronounced characteristic peak at 30.6°. This distinctive peak arises from the potent surface polarity of silicate, leading to the formation of agglomerates within the coating. Upon the addition of urea and melamine in ASM_7_U_7_, there was a partial enhancement in the intensity of peaks at 22.1° and 26.1°. The absence of a characteristic peak at 30.6° in the ternary synergistic flame-retardant coating indicates the formation of a complex between urea and silanol. This complex reduced the linearity of the silicon skeleton, and concurrently, hydrogen bonding between the amine group in melamine and the silicon skeleton hindered the polymerization of sodium silicate. These collaborative effects contributed to a reduction in the characteristic peaks of sodium silicate in the ternary synergistic flame-retardant coating, underscoring the beneficial impact of incorporating urea and melamine in the process of mitigating sodium silicate agglomeration in the coating.

### 2.6. Particle Size Analysis

The dispersion of flame retardants within coatings plays a pivotal role in influencing the flame retardant’s performance. The presence of fewer and smaller agglomerates signifies enhanced dispersion, resulting in a more uniform distribution of flame retardants within the coating. Therefore, achieving optimal dispersion of flame retardants in coatings is an indispensable prerequisite for crafting flame-retardant coatings with superior flame-retardant properties. The results of particle size analysis reveal that the particle sizes of A, AS, and the ternary synergistic flame-retardant coating (ASM_7_U_7_) were 102.2 nm, 113.1 nm, and 104.9 nm, respectively. Notably, the particle size of the sodium silicate flame-retardant coating was the largest, while that of the ternary synergistic flame-retardant coating was reduced by 8.2 nm, representing a decrease of 7.3%. This reduction in particle size in the ternary synergistic flame-retardant coating, attributed to the addition of melamine and urea modifiers, signifies an improvement in the dispersion performance of sodium silicate within the waterborne acrylic acid coating. This modification effectively overcomes the challenge of agglomeration, contributing to a more homogeneous distribution of flame retardants within the coating.

### 2.7. Mechanism of Synergistic Flame Retardancy in Sodium Silicate/Melamine/Urea Ternary System

**Figure 5:**
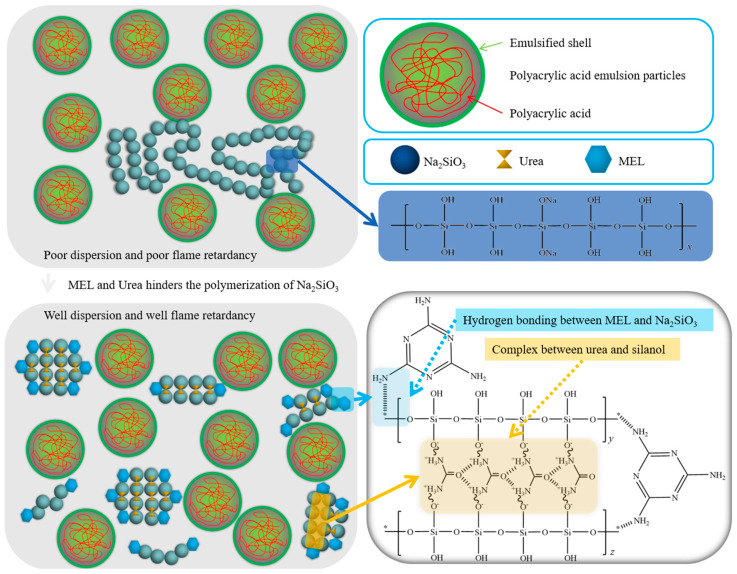
Mechanism of synergistic modification of sodium silicate with melamine and urea.

Based on the research conducted by Yu et al. [32], it was found that novel organic–inorganic composite materials could be prepared through the crosslinking of ion oligomers using a copolymerization approach. The copolymers synthesized in their work exhibited a continuous mixed network without distinct interface boundaries. The ion binding effect significantly enhanced the mechanical strength during crosslinking, surpassing the performance of other PAM-based composite materials. Inspired by the organic–inorganic copolymerization research by Yu et al., we propose a strategy for modifying sodium silicate by incorporating melamine and urea. This modification aims to improve its dispersion performance, overcome agglomeration issues, and simultaneously achieve synergistic flame retardancy. The mechanism is illustrated in Figure 5.

When unmodified sodium silicate is introduced into waterborne acrylic acid coatings, it typically exists in a long-chain form. Free sodium silicate gradually aggregates to form large molecules. Simultaneously, the hydrophilic hydroxyl groups on the surface of sodium silicate continuously absorb moisture from the coating, accelerating agglomeration and significantly reducing its dispersion performance in the coating. This compromises the ability of flame-retardant coatings to form a uniform heat-insulating layer on the material surface, leading to a decline in flame-retardant performance. When sodium silicate modified with melamine and urea is applied to prepare ternary synergistic flame-retardant coatings, the urea forms a complex with silanol, reducing the linearity of the silicon skeleton. Simultaneously, the amine groups in melamine form hydrogen bonds, hindering the polymerization of sodium silicate and making it resistant to agglomeration. This substantial improvement in dispersion performance in waterborne acrylic acid coatings contributes to the elevated flame-retardant performance of the coating.

In this context, sodium silicate primarily functions as a flame retardant by forming a stable silicon-containing char layer, preventing the outward escape of combustible substances generated during combustion. It also acts as a thermal and oxygen barrier. Melamine and urea, as modifiers and synergistic flame retardants, enhance the dispersion of sodium silicate in waterborne acrylic acid and assist in the formation of a silicon-containing char layer by generating nitrogen gas. This synergistic action results in the formation of a protective layer of silicide, effectively achieving the goal of flame retardancy.

### 2.8. Ignition Time and Flame Height

**Figure 6:**
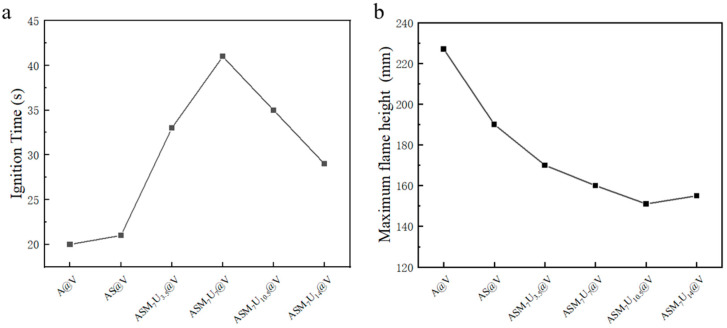
Ignition time and flame height After ignition of poplar veneers. (**a**) Ignition time; (**b**) flame height after ignition.

The ignition time and flame height of A@V, AS@V, and ternary synergistic flame-retardant poplar veneers (ASM_7_U_3.5_@V, ASM_7_U_7_@V, ASM_7_U_10.5_@V, ASM_7_U_14_@V) are depicted in Figure 6. From Figure 6a, it can be observed that the ignition time of the flame-retardant poplar veneer surpassed that of the non-flame-retardant poplar veneer. Notably, the ignition time of the ternary synergistic flame-retardant poplar veneers exceeded that of sodium silicate flame-retardant poplar veneer, ranging from a minimum of 29 s to a maximum of 41 s. These values represent 138% and 195% of the ignition time of the sodium silicate flame-retardant poplar veneer (21 s), respectively. The longest ignition time occurred when both melamine and urea had a mass percentage of 7%. Consequently, the flame-retardant performance of sodium silicate was significantly enhanced following synergistic modification with melamine and urea, with the optimal performance observed when both melamine and urea had a mass percentage of 7% (ASM_7_U_7_@V). This indirectly verifies that the addition of modifiers substantially improves the dispersibility of sodium silicate flame retardants in water-based acrylic coatings.

From Figure 6b, it is evident that the flame height of flame-retardant poplar veneer after ignition is less than that of non-flame-retardant poplar veneer. Specifically, the flame height of ternary synergistic flame-retardant poplar veneers is lower than that of the sodium silicate flame-retardant poplar veneer, with a minimum of 151 mm and a maximum of 170 mm. These values represent 79% and 89% of the flame height of sodium silicate flame-retardant poplar veneer after ignition (190 mm), respectively. Therefore, the flame-retardant performance of sodium silicate is significantly improved after synergistic modification with melamine and urea. This indirectly supports the notion that the addition of modifiers greatly enhances the dispersibility of sodium silicate flame-retardant in water-based acrylic coatings.

**Figure 7:**
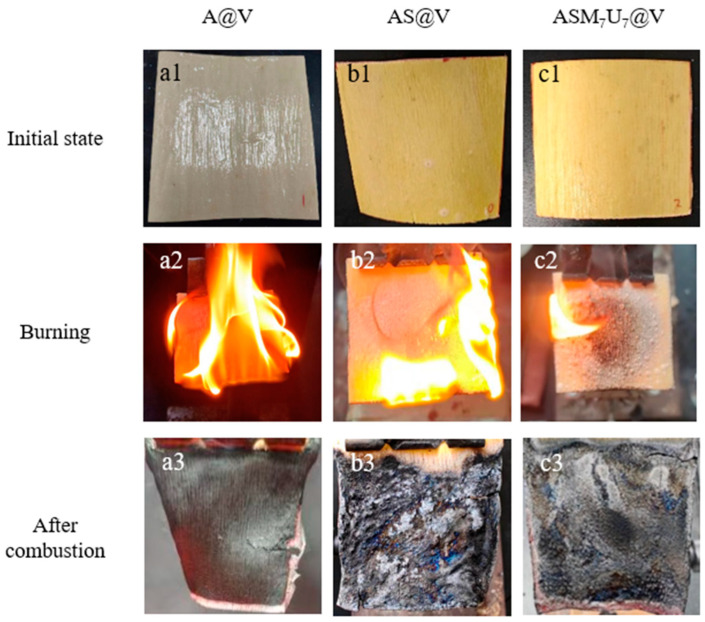
Photos of poplar veneers before and after ignition. (**a1**–**a3**) photos of A@V before and after ignition; (**b1**–**b3**) photos of AS@V before and after ignition; (**c1**–**c3**) photos of ASM_7_U_7_@V before and after ignition.

The visual representation of the states before and after ignition for A@V, AS@V, and ASM_7_U_7_@V is illustrated in Figure 7. Examining the figure, the coating surface of the non-flame-retardant poplar veneer (a1) exhibited a white color, while the coating surfaces of the flame-retardant poplar veneers (b1, c1) displayed a yellow hue. This alteration in color indicates the impact of flame-retardant coatings on the surface appearance of poplar veneer, while preserving the distinct wood texture.

The flame height of the non-flame-retardant poplar veneer after ignition (a2) was higher, with a rapid flame spread. Conversely, the flame height of the flame-retardant poplar veneers after ignition (b2, c2) was lower, and the flames were more subdued. Particularly noteworthy was the presence of white bubbles on the surface of the ternary synergistic flame-retardant poplar veneer (c2). This occurrence can be attributed to the combustion-induced decomposition of melamine and urea, generating nitrogen gas. This nitrogen gas combined with the carbon source, sodium silicate, producing layered silica. The resulting bubbles caused the coating to expand, creating a barrier against oxygen, thereby reducing the flame spread speed, prolonging the combustion time, and lowering the flame height.

Additionally, the non-flame-retardant poplar veneer’s char layer (a3) was thin and presented as a light gray residue. In contrast, the sodium silicate flame-retardant poplar veneer’s char layer (b3) was thicker, harder, and appears as black block-like residues. The char layer of the ternary synergistic flame-retardant poplar veneer (c3) was the thickest and hardest, displaying intact, black, block-like residues. This indicates a significant enhancement in the flame retardancy of sodium silicate through synergistic modification with melamine and urea. Importantly, it indirectly highlights that the addition of modifying agents substantially improved the dispersion performance of the sodium silicate flame retardant in waterborne acrylic acid coatings.

In summary, the ternary synergistic flame-retardant poplar veneer demonstrated superior flame-retardant performance, meeting the stringent requirements outlined in both the Chinese national standards “Classification of Combustion Performance of Building Materials and Products” (GB 8624-2012) and “Flame Retardant Plywood” (GB/T 18101-2013), achieving the coveted flame-retardant B1 classification. The above results and discussion are all based on experiments conducted on poplar veneer, so they may not be applicable to other materials such as steel and textiles, but they still have certain reference value.

### 2.9. Limiting Oxygen Index (LOI) Analysis

**Figure 8:**
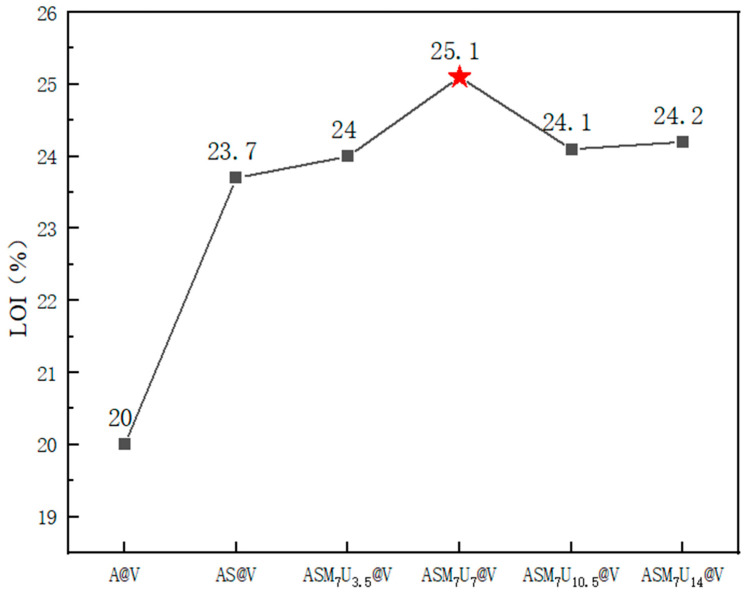
Changes in limiting oxygen index before and after flame retardancy of poplar veneer. (The red star marks the highest limit oxygen index in each group).

The limiting oxygen index (LOI) is a critical parameter that represents the minimum concentration of oxygen, expressed as a percentage by volume, required for a material to sustain candle-like flaming combustion under specified conditions in a mixture of oxygen and nitrogen. A higher LOI signifies a reduced susceptibility to combustion, indicating improved flame-retardant performance [33,34]. The LOI values for A@V, AS@V, and ternary synergistic flame-retardant poplar veneer (ASM_7_U_3.5_@V, ASM_7_U_7_@V, ASM_7_U_10.5_@V, ASM_7_U_14_@V) are depicted in Figure 8. From Figure 8, non-flame-retardant poplar veneer exhibits an LOI of 20%, categorizing it as a flammable material without flame retardancy. In comparison, sodium silicate flame-retardant poplar veneer demonstrates an LOI of 23.7%, surpassing that of its non-flame-retardant counterpart. The LOI values for flame-retardant poplar veneer, prepared with various ratios of ternary synergistic flame-retardant coating, consistently exceed that of sodium silicate flame-retardant poplar veneer. This signifies a notable enhancement in flame-retardant performance following the collaborative modification of sodium silicate with melamine and urea. Among these, ASM_7_U_7_@V stands out with the highest LOI value, reaching 25.1%. According to the GB/T 2406.2-2009 standard, materials with LOI values falling within the range of 21% to 27% are classified as slow-burning flame retardants [35,36]. Hence, the flame-retardant poplar veneer was appropriately categorized as slow-burning flame-retardant material. The above results and discussion are all based on experiments conducted on poplar veneer, so they may not be applicable to other materials such as steel and textiles, but still have certain reference value.

### 2.10. Thermogravimetric (TG and DTG) Analysis

**Figure 9:**
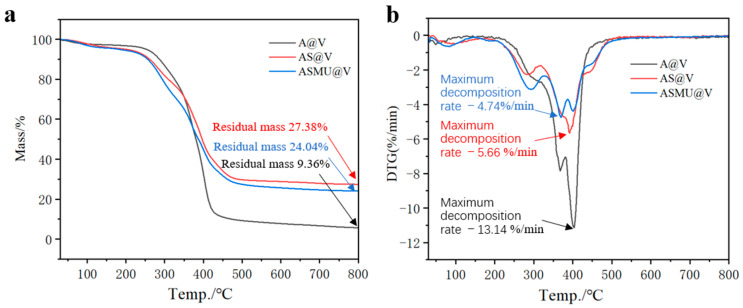
Thermogravimetric analysis of poplar veneer before and after flame retardancy. (**a**) TG; (**b**) DTG.

Figure 9 presents the TG curves and DTG curves for A@V, AS@V, and ASM_7_U_7_@V. Analyzing the TG curve (Figure 9a), it is evident that the residue mass fractions for the sodium silicate flame-retardant poplar veneer and the ternary synergistic flame-retardant poplar veneer were 27.38% and 24.04%, respectively. These values represent substantial increases of 192.52% and 156.83%, respectively, compared to the 9.36% residue mass fraction of non-flame-retardant poplar veneer. The significant elevation in residue mass fraction indicates improved flame retardancy [37,38]. Notably, the slightly lower residue mass fraction in ternary synergistic flame-retardant poplar veneer compared to sodium silicate flame-retardant poplar veneer can be attributed to the gas generation from melamine and urea at high temperatures. Therefore, both the sodium silicate flame-retardant coating and the ternary synergistic flame-retardant coating contributed to enhancing the flame retardancy of non-flame-retardant poplar veneer. Examining the DTG curve (Figure 9b), the peak decomposition rates of the sodium silicate flame-retardant poplar veneer and the ternary synergistic flame-retardant poplar veneer were 5.66%/min and 4.74%/min, respectively. These values represent only 43.07% and 36.07% of the 13.14%/min peak decomposition rate of non-flame-retardant poplar veneer, indicating a substantial reduction in the decomposition rate. Remarkably, the ternary synergistic flame-retardant poplar veneer showed the most significant reduction in the peak decomposition rate. Therefore, ternary synergistic flame-retardant poplar veneer exhibited superior flame retardancy [39,40], with the addition of melamine and urea-modifying agents significantly improving the flame retardancy of the sodium silicate flame-retardant coating.

### 2.11. Cone Calorimeter (CONE) Analysis

**Figure 10:**
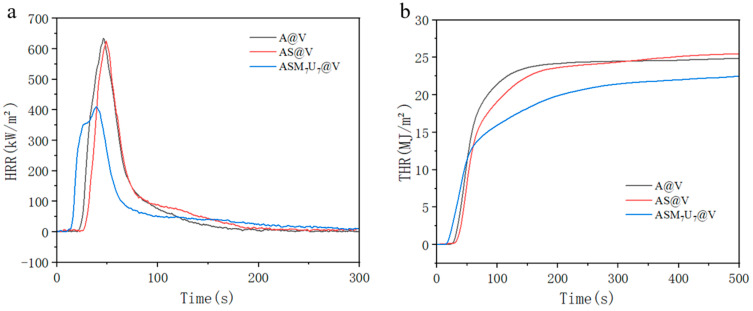
Flame retardancy performance before and after burning poplar veneer. (**a**) HRR; (**b**) THR.

The cone calorimeter test results for A@V, AS@V, and ASM_7_U_7_@V are presented in Figure 10. In Figure 10a, the heat release rate (HRR) curve reveals that the ternary synergistic flame-retardant poplar veneer exhibited the lowest peak heat release rate, reduced by 35.3% and 34.3% compared to non-flame-retardant poplar veneer and sodium silicate flame-retardant poplar veneer, respectively. The peak heat release rate of sodium silicate flame-retardant poplar veneer was only marginally lower, decreasing by 1.5% compared to the non-flame-retardant poplar veneer. This highlights the substantial enhancement of flame retardancy in the sodium silicate flame-retardant coating through the inclusion of melamine and urea-modifying agents [41,42]. In Figure 10b, the total heat release (THR) curve indicates that the ternary synergistic flame-retardant poplar veneer had the lowest total heat release, decreasing by 11.3% and 9.5% compared to the non-flame-retardant poplar veneer and sodium silicate flame-retardant poplar veneer, respectively. Conversely, the total heat release of the sodium silicate flame-retardant poplar veneer increased by 2% compared to the non-flame-retardant poplar veneer. This underscores the significant improvement in the flame retardancy of the sodium silicate flame-retardant coating with the addition of melamine and urea-modifying agents.

**Figure 11:**
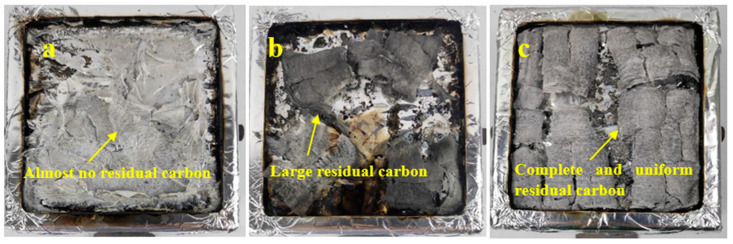
Residual char photographs after burning poplar veneer. (**a**) A@V char; (**b**) AS@V char; (**c**) ASM_7_U_7_@V char.

Figure 11 displays photographs of the residual char after burning A@V, AS@V, and ASM_7_U_7_@V. Observing Figure 11a, the residual material after burning non-flame-retardant poplar veneer mainly consisted of grayish-white powder, with little to no formation of charcoal blocks. Figure 11b illustrates that the residual material after burning sodium silicate flame-retardant poplar veneer partly contained grayish-white powder and partly exhibited grayish-black charcoal blocks, with noticeable cracking in the charcoal blocks. In contrast, Figure 11c shows that the residual material after burning ternary synergistic flame-retardant poplar veneer predominantly comprised grayish-black charcoal blocks, with fewer instances of cracking. Consequently, the flame retardancy of the ternary synergistic flame-retardant poplar veneer surpassed that of the non-flame-retardant poplar veneer and the sodium silicate flame-retardant poplar veneer [43]. This underscores the significant improvement in the flame retardancy of sodium silicate, indirectly indicating the modifying agents’ ability to substantially enhance the dispersion performance of the sodium silicate flame retardant in waterborne acrylic acid coatings. The above results and discussion are all based on experiments conducted on poplar veneer, so they may not be applicable to other materials such as steel and textiles, but still have certain reference value.

### 2.12. UL-94 Vertical Burning Test Results Analysis

**Figure 12:**
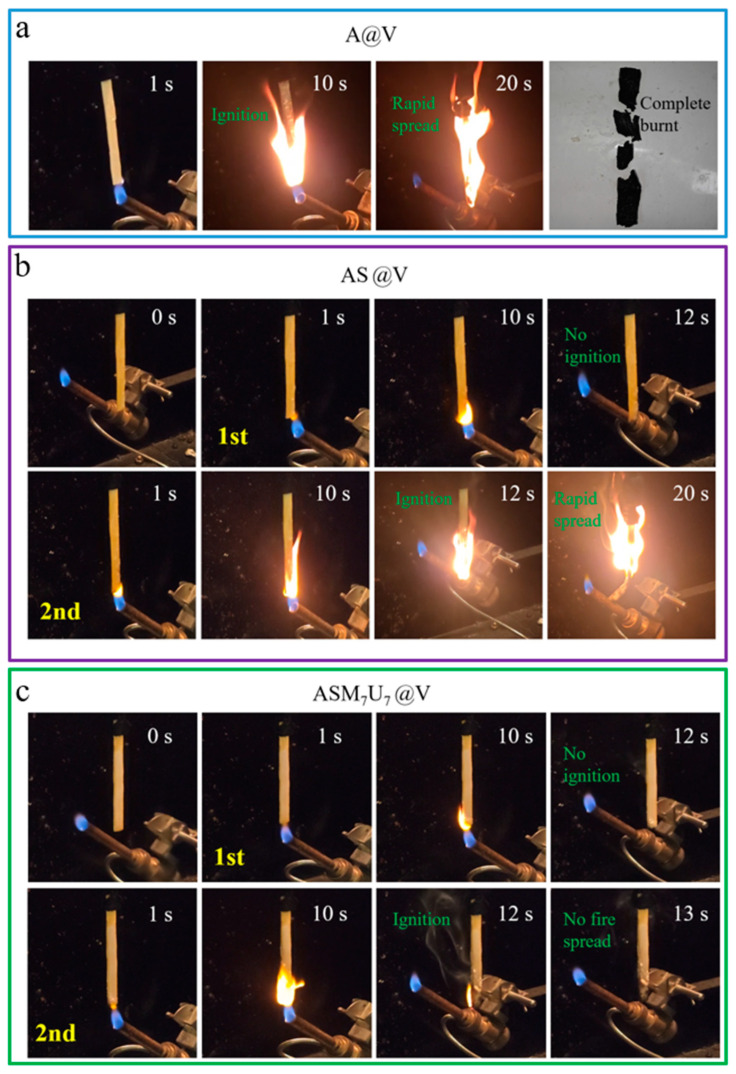
Video snapshot from UL-94 test. (**a**) A@V; (**b**) AS@V; (**c**) ASM_7_U_7_@V.

The UL-94 test results for A@V, AS@V, and ASM_7_U_7_@V are presented in Figure 12. The results indicate that A@V, AS@V, and ASM_7_U_7_@V all showed no dripping phenomenon. Specifically, A@V burned completely within a short time. AS@V was quickly extinguished after the first ignition, but flames spread upon the second ignition and could not be extinguished until complete combustion. ASM_7_U_7_@V was quickly extinguishes after the first ignition, and was extinguished 2.9 s after the second ignition. A@V and AS@V failed to meet the lowest flame-retardant rating in the UL-94 standard, while ASM_7_U_7_@V exhibited excellent flame retardancy and achieved a V-0 rating. Consequently, the ternary synergistic flame-retardant coating significantly enhanced the flame-retardant performance of poplar veneer and outperformed AS@V, demonstrating that the addition of melamine and urea modifiers substantially improved the flame-retardant properties of sodium silicate flame-retardant coatings. The above results and discussion are all based on experiments conducted on poplar veneer, so they may not be applicable to other materials such as steel and textiles, but still have certain reference value.

## 3. Materials and Methods

### 3.1. Materials

Waterborne acrylic coatings (C_3_H_4_O_2_, E0504) were purchased from Shenzhen Jitian Chemical Co., Ltd., Shenzhen, China. Sodium metasilicate nonahydrate (Na_2_SiO_3_·9H_2_O), melamine (C_3_H_6_N_6_), and urea (CH₄N₂O) were analytically pure and produced by Sinopharm Chemical Reagent Co., Ltd., Shanghai, China. Deionized water was made by the laboratory. Poplar veneers were cut into sizes of 400 mm × 2 mm × 400 mm (chord × radial × axial) with moisture contents of 10 ± 2%; they were produced by Linyi Poplar Wood Veneer Factory, Linyi, China.

### 3.2. Methods

#### 3.2.1. Preparation of Flame-Retardant Coatings

In flame-retardant coatings, the mass ratio of flame-retardant to waterborne acrylic coating was set at 3:7. The waterborne acrylic coating without the addition of flame-retardant, referred to as the non-flame-retardant coating (A), was designated as the first control group. Within the waterborne acrylic coating, a certain amount of sodium silicate flame retardant was added without the use of urea or melamine modifiers to prepare the coating, referred to as the sodium silicate flame-retardant coating (AS). This was designated as the second control group. For the sodium silicate flame-retardant coating, coatings were prepared by adding urea and melamine modifiers. This was referred to as the sodium silicate/urea/melamine ternary synergistic flame-retardant coating, abbreviated as the ternary synergistic flame-retardant coating (ASMU). The ternary synergistic flame-retardant agent is prepared by mixing sodium silicate flame retardant, urea modifier, and melamine modifier in a certain ratio. The mass percentage of melamine was set at 7%, and when the mass percentages of urea were 3.5%, 7%, 10.5%, and 14%, the corresponding ternary synergistic flame-retardant coatings were labeled as ASM_7_U_3.5_, ASM_7_U_7_, ASM_7_U_10.5_, and ASM_7_U_14_, respectively. During the preparation of the ternary synergistic flame-retardant agent, waterborne acrylic coatings, sodium silicate, melamine, and urea were separately weighed according to the aforementioned ratios and placed into a beaker. The beaker containing the waterborne acrylic coatings was then placed into a constant temperature oil bath at 40 °C. Sodium silicate was added under stirring conditions and stirred for 10 min. Subsequently, melamine and urea were added separately and stirred for 30 min. After the coating was uniformly mixed, the sodium silicate/urea/melamine ternary synergistic flame-retardant coating was obtained.

#### 3.2.2. Viscosity Test of Coatings

The viscosity of the coating samples was assessed utilizing a QSG-type format viscometer. The ascent time of the bubble column was observed, and viscosity was determined based on a time–viscosity correlation table. Coatings A, AS, ASM_7_U_3.5_, ASM_7_U_7_, ASM_7_U_10.5_, and ASM_7_U_14_ were poured into the viscosity tube, reaching approximately the 100 mm mark, then sealed with a plug and immersed in 25 °C water. After 10 min, the liquid level was adjusted to 100 mm, sealed at the 108 mm mark, and continued in a 25 °C constant water bath. Another 10 min later, the viscosity tube was swiftly inverted, and the time taken in the 25 °C water bath for the bubble to reach the top of the tube was measured.

#### 3.2.3. Preparation of Flame-Retardant Poplar Veneer

Poplar veneer, initially 400 mm × 2 mm × 400 mm, was trimmed to specimens of 65 mm × 2 mm × 65 mm. Coatings A, AS, ASM_7_U_3.5_, ASM_7_U_7_, ASM_7_U_10.5_, and ASM_7_U_14_ were uniformly applied to the veneer surface along the grain direction with a brush. During the coating process, an even coat was first applied with a fine-bristle brush, followed by another coat with a roller brush. The amount of coating was rigorously controlled using an electronic balance, and the coating was briefly dried in an oven before repeating the process for a second coat using the same method. This ensured that the coating amount reached 300 g/m^2^ while achieving uniformity. Following an 8 h drying period at 50 °C and drying at ambient temperature for 48 h, non-flame-retardant poplar veneer (A@V), sodium silicate flame-retardant poplar veneer (AS@V), and ternary synergistic flame-retardant poplar veneer (ASM_7_U_3.5_@V, ASM_7_U_7_@V, ASM_7_U_10.5_@V, ASM_7_U_14_@V) were prepared.

#### 3.2.4. Paint Film Hardness Test

The hardness of the paint film on flame-retardant poplar veneer samples was determined using a QHQ-A portable pencil scratch tester. According to ISO 15184:2010, various hardness pencils were employed for testing, and the pencil that produced scratches not exceeding 3 mm was identified as the coating hardness [44,45].

#### 3.2.5. Paint Film Adhesion Test

Using a hundred-grid knife with a model number of QFH-A, the flame-retardant poplar veneer samples were placed on a sufficiently rigid surface. With the hundred-grid knife handle held by hand, the multi-blade cutting knife was positioned perpendicular to the sample plane. Applying uniform pressure and a cutting speed of 20–50 mm/s, a 10 × 10 grid of 1 mm×1 mm small squares was cut into the test sample surface. Each line touched the material. A 3M 610 adhesive tape with an adhesion force of 350–400 g/cm^2^ was used for three grid pull tests, and we observed film detachment at the edges and intersections of the scored lines [46,47].

#### 3.2.6. Scanning Electron Microscope (SEM)

The coating surface and cross-sectional microstructures of the samples were investigated using a field emission SEM analyzer (HITACHI SU8010) with an acceleration voltage of 3 kV. All the samples were sputter-coated with 4 nm of platinum layer prior to SEM imaging.

#### 3.2.7. Energy-Dispersive X-ray Spectroscopy (EDX)

Elemental analysis of the samples was conducted using an energy-dispersive X-ray spectrometer (HITACHI SU8010) to detect the relative contents of C, O, N, Na, and Si elements. EDX spectra were collected on selected areas (5 μm × 5 μm) at a 10 kV acceleration voltage.

#### 3.2.8. X-ray Diffraction (XRD)

X-ray diffraction (Bruker D2) was employed to test and analyze the phase composition and crystallinity of the samples. In addition to testing AS and ASM_7_U_7_ coatings, a sodium silicate solution was tested as a control group (S). The scanning range and speed of the X-ray diffraction instrument were 10–60° (2*θ*) and 5°/min, respectively. The results were analyzed in Jade software (Release 6.5.26 @ 07/02/05) to discuss the phase composition and crystallinity of the samples.

#### 3.2.9. Particle Size Test

A ZETA potential and a particle size analyzer (Bruker 90Plus PALS) were used to assess the particle sizes of the prepared coating samples under different conditions. The particle size of sodium silicate was evaluated to analyze its dispersion in the coating.

#### 3.2.10. Ignition Test

Reference to the Chinese national standard “Classification of Combustion Performance of Building Materials and Products” (GB 8624-2012) and “Non-flammable Plywood” (GB/T 18101-2013), the ignition test was designed to assess the flame retardancy of poplar veneer [35,48]. The following specimens were tested: untreated coated poplar veneer (V, control group), A@V, AS@V, and ternary synergistic flame-retardant poplar veneer (ASM_7_U_3.5_@V, ASM_7_U_7_@V, ASM_7_U_10.5_@V, ASM_7_U_14_@V). The same test number was repeated three times, and the results were averaged. The experimental setup included an iron frame stand, a support, an alcohol lamp, a clamp, and a metal ruler. The clamp was fixed at a height of 310 mm, and the lifting platform was adjusted to a height of 150 mm, ensuring stable external flame heating of the veneer. In a wind-free environment, we ignited the alcohol lamp and placed it directly below the veneer’s center for heating. We used a clamp to hold one side perpendicular to the veneer grain and started the stopwatch. We observed the flame below the veneer; when the flame spread around the veneer, we removed the alcohol lamp and stopped the timer. We observed the burning situation, and if the veneer self-extinguished, we continued heating with the alcohol lamp and continued timing. When removing the alcohol lamp for the last time, if the veneer did not extinguish without a fire source, that timing was the ignition time of the veneer. Additionally, we recorded the highest flame height during the combustion process.

#### 3.2.11. Limiting Oxygen Index (LOI)

Wooden strips with dimensions of 120 mm × 2 mm × 10 mm were prepared from samples including A@V, AS@V, and ternary synergistic flame-retardant poplar veneer (ASM_7_U_3.5_@V, ASM_7_U_7_@V, ASM_7_U_10.5_@V, ASM_7_U_14_@V). The oxygen index content was determined using an oxygen index tester (LOI). Each test was replicated six times under the same test number, and the results were averaged.

#### 3.2.12. Thermogravimetric (TG)

The thermal stability of waterborne acrylic flame-retardant coatings before and after flame retardancy was investigated using a thermogravimetric analyzer (TG) with N_2_ as a protective gas. The temperature range was maintained between 35~800 °C, with a controlled heating rate of 20 °C/min.

#### 3.2.13. Cone Calorimeter (CONE)

The flame-retardant performance of the samples was assessed using a cone calorimeter (CONE), characterizing a size of 100 mm × 2 mm × 100 mm sample of retardant poplar veneer to measure parameters such as the heat release rate (HRR) and total heat release (THR). The heat radiation power was set at 50 kW/m^2^, and each test number underwent three repetitions.

#### 3.2.14. UL-94 Vertical Burning Test

We used the UL-94 vertical burning test to assess the UL-94 ratings of A@V, AS@V, and ASM_7_U_7_@V, with sample dimensions of 125 mm × 2 mm × 13 mm. The testing standard was GB/T2408-2008.

## 4. Conclusions

This work employed sodium silicate as a flame retardant, with urea and melamine serving as modifying and synergistic agents, to prepare a ternary synergistic water-borne acrylic acid flame-retardant coating which was applied to the surface of poplar veneer. The properties and mechanisms of the flame-retardant coating were investigated. The main results and conclusions are as follows:

(1) Compared with waterborne acrylic acid-based sodium silicate flame-retardant coatings, the ternary synergistic waterborne acrylic acid flame-retardant coating (sodium silicate/urea/melamine) exhibited reduced viscosity, decreased particle size, and more uniform dispersion of the flame retardant, preventing agglomeration.

(2) The ternary synergistic flame-retardant poplar veneer coating demonstrated uniform distribution of Si elements and an absence of crystalline and agglomeration phenomena, achieved HB-level paint film hardness, and exhibited 4B-level paint film adhesion. These characteristics indicate favorable physical and mechanical properties. The addition of melamine and urea as modifying agents significantly improved the dispersion of sodium silicate in the waterborne acrylic acid coating, overcoming agglomeration issues.

(3) Compared with non-flame-retardant poplar veneer, the ternary synergistic flame-retardant poplar veneer showed a significantly prolonged ignition time, a substantial reduction in flame height after ignition, a noticeable increase in the limiting oxygen index, and a substantial decrease in the peak heat release rate. The thickness and hardness of the residual carbon layer substantially increased, resulting in more complete carbon residue. The best flame-retardant performance was observed when the mass percentages of melamine and urea were both 7%.

(4) Melamine and urea, as modifying agents, not only significantly improved the dispersion of sodium silicate in waterborne acrylic acid, but also assisted in the formation of a silicon-containing char layer, achieving synergistic flame retardancy.

In summary, this work explored an efficient and uniform dispersion method for inorganic flame-retardants in organic coatings. This improvement led to an enhanced flame-retardant performance, providing theoretical reference for the research and application of novel flame-retardant coatings.

## Figures and Tables

**Table 1:** Paint film hardness and adhesion of untreated poplar veneer and flame-retardant-treated poplar veneer.

Group	A@V	AS@V	ASM_7_U_3.5_@V	ASM_7_U_7_@V	ASM_7_U_10.5_@V	ASM_7_U_14_@V
Paint film hardness	HB	HB	B	HB	HB	HB
Paint film adhesion	4B	4B	4B	4B	4B	4B

## Data Availability

The data that has been used is confidential.
